# Associations between fitness social media exposure and exercise adherence: dual pathways via parasocial relationships and body image pressure

**DOI:** 10.3389/fpsyg.2026.1860956

**Published:** 2026-07-02

**Authors:** Chongyang Li, Yaya Shi

**Affiliations:** 1 Graduate School, Jilin Sport University, Changchun, China; 2 Department of Physical Education, Yan’an University, Yan’an, China

**Keywords:** body image pressure, exercise adherence, exercise self-efficacy, fitness social media, parasocial relationship

## Abstract

**Introduction:**

Although fitness social media has emerged as a primary source of exercise information and health advice, the mechanisms linking such exposure to exercise adherence remain poorly understood. This study develops and tests a dual-pathway framework examining how fitness social media exposure is associated with exercise adherence among recent content consumers.

**Methods:**

Cross-sectional data from 575 respondents were analyzed using partial least squares structural equation modeling (PLS-SEM) to evaluate variable net effects, complemented by fuzzy-set qualitative comparative analysis (fsQCA) to identify multi-condition configurations associated with high exercise adherence.

**Results:**

Fitness social media exposure was associated with parallel positive and negative psychological processes, but the positive pathway showed substantially greater explanatory power. Specifically, media exposure was significantly and positively associated with both parasocial relationships and physical appearance comparison. The positive pathway through parasocial relationships, exercise self-efficacy, and exercise identity was fully supported, whereas the negative pathway through body image pressure was only partially supported, as the effect of body surveillance on social physique anxiety was non-significant.

**Discussion:**

Exercise self-efficacy and exercise identity emerged as key proximal variables in the net-effect model and as stable core conditions in configurations associated with high exercise adherence. These findings suggest that relational and self-regulatory mechanisms are central to understanding exercise adherence among fitness content consumers and may outweigh the negative role of appearance pressure.

## 1 Introduction

Fitness social media has become a primary conduit for exercise advice, training information, and health knowledge (Dennehy et al., 2024; Powell and Pring, 2024). Because exercise adherence is critical for translating physical activity into long-term health benefits (André et al., 2024), public health research increasingly prioritizes sustained behavioral engagement over mere initiation. Consequently, the impact of fitness social media exposure on exercise adherence warrants rigorous investigation, particularly given conflicting findings in the literature. While some evidence indicates that such exposure enhances exercise intention, training readiness, and health behavior participation (Yang, 2025; Breves et al., 2025a), other research demonstrates that it exacerbates appearance-based normative pressure and negative self-evaluations, undermining potential benefits (Liu, 2025). This discrepancy suggests that the relationship between media exposure and adherence is not unidirectionally facilitative; rather, it operates through parallel positive and negative psychological mechanisms.

Regarding the positive mechanisms, frequent exposure to fitness content supplies users with resources for emotional connection, behavioral modeling, and self-definition. Repeated interaction with specific fitness influencers fosters parasocial relationships characterized by a high degree of psychological realism (Feng et al., 2025; Breves et al., 2025a). Despite lacking real-world reciprocity, these one-sided relationships position media figures as credible, approachable training referents. This modeling process cultivates users’ belief in their capacity to execute training tasks, thereby enhancing exercise self-efficacy. Concurrently, sustained exposure facilitates the internalization of a “regular exerciser” identity within the user’s self-concept, establishing a stable exercise identity. Given that both exercise self-efficacy and identity strongly predict sustained exercise behavior (Li et al., 2024; Rhodes et al., 2025), these factors—driven by parasocial relationships—jointly constitute a critical positive pathway linking fitness social media exposure to long-term exercise adherence.

Conversely, fitness social media exposure frequently precipitates adverse psychological outcomes. Because such content is predominantly visual, emphasizing idealized physiques, low adiposity, and aesthetic results, it inherently triggers physical appearance comparisons among consumers (Breves et al., 2025a; Brasil et al., 2024; Ladwig et al., 2024). Chronic comparison with these idealized images diverts attention from the intrinsic benefits of exercise toward conformity with external aesthetic standards. This cognitive shift exacerbates body surveillance, characterized by persistent hypervigilance regarding physical appearance and external judgments. Consequently, this externally referenced schema generates heightened distress in exercise or body display contexts, precipitating social physique anxiety (Moffitt, 2024; Liu, 2025). Under these conditions, exercise is reconceptualized as a compulsory response to appearance pressure rather than a proactive strategy for competence enhancement and health management, ultimately eroding behavioral autonomy, intrinsic enjoyment, and sustained participation.

Although existing literature documents both the facilitative and deleterious effects of fitness social media, previous research predominantly isolates these mechanisms into unidirectional models. These investigations either highlight mobilization for health behaviors or underscore risks associated with body image and anxiety (Powell and Pring, 2024; Yang, 2025; Liu, 2025; Breves et al., 2025a; Breves et al., 2025b). Comprehensive frameworks simultaneously evaluating positive psychological facilitators and negative body image pressures remain scarce. To address this gap, a dual pathway model is tested to determine the relative explanatory power of parasocial relationships, exercise self-efficacy, exercise identity, physical appearance comparison, body surveillance, and social physique anxiety. Methodologically, an integration of PLS-SEM and fsQCA provides a robust framework: PLS-SEM isolates net effects and identifies the primary mechanisms, while fsQCA elucidates multiple condition configurations associated with high exercise adherence and causal asymmetry. Ultimately, rather than reducing digital fitness exposure to a singular influence, this framework conceptualizes the exposure and adherence relationship as a complex structure where parallel positive and negative processes operate with asymmetric strength.

## 2 Literature review and theoretical background

### 2.1 Theoretical foundations of the dual pathways linking fitness social media exposure to exercise adherence

#### 2.1.1 The parasocial relationship perspective, social cognitive theory, and identity theory

Horton and Wohl (1956) introduced the concept of parasocial relationships to describe the unilateral emotional intimacy audiences develop with media figures through sustained exposure. Within fitness social media, this manifests as prolonged user engagement with specific influencers’ training routines, dietary regimens, and physiological transformations. Repeated interactions—such as viewing, liking, and commenting—cultivate familiarity and trust. As exposure frequency and perceived intimacy increase, this unilateral bond deepens (Rubin and McHugh, 1987). Contemporary research confirms that the inherent interactivity and companionship of digital fitness content facilitate these parasocial bonds, with repeated exposure solidifying the psychological connection between audiences and influencers (Feng et al., 2025; Breves et al., 2025b). When influencers are perceived as ubiquitous and credible, their exercise recommendations are more readily integrated into users’ daily practices, establishing an emotional foundation for exercise readiness (Yang, 2025).

Building upon this emotional foundation, social cognitive theory elucidates how parasocial ties translate into actionable beliefs. Bandura (2001) posited that learning extends beyond experiential trial; individuals form cognitive representations by observing behavioral models and their self-regulatory processes. Self-efficacy serves as the pivotal mechanism converting observational learning into sustained action. Fitness influencers provide concrete, highly visualized vicarious experiences by documenting their training routines and physical progression via digital platforms. Crucially, demonstrating adherence despite fatigue or emotional depletion enhances users’ perceived competence to overcome exercise barriers. Because exercise self-efficacy robustly predicts subsequent participation (McAuley, 1993) and behavioral persistence (Li et al., 2024), fitness influencers function as direct observational models who stimulate both initial behavioral imitation and long-term habit maintenance through heightened self-efficacy.

However, initial action beliefs alone insufficiently explain long-term adherence; identity theory provides a structural mechanism for behavioral routinization. Burke (2004) conceptualized that individuals align their behaviors with specific social roles to preserve self-concept coherence. In fitness contexts, this involves internalizing the role of a “regular exerciser” as a central component of self-definition (Anderson and Cychosz, 1994). For fitness social media users, continuous interaction and observational learning transcend the transient imitation of isolated workouts. Coupled with repeated emotional investment and mastery experiences, these processes transform exercise from an extrinsic activity into an intrinsic identity attribute. Rhodes et al. (2025) confirmed that physical activity identity correlates stably with actual behavior and, synergistically with self-regulatory mechanisms, sustains long-term adherence. Synthesizing these perspectives: parasocial relationships establish emotional bonds, self-efficacy cultivates actionable beliefs, and exercise identity internalization ensures behavioral persistence. Together, these interconnected frameworks constitute the positive psychological mechanisms driving exercise adherence in digital fitness contexts.

#### 2.1.2 Social comparison theory and objectification theory

Festinger (1954) posited that individuals inherently evaluate their abilities and status through interpersonal comparison. When targets are perceived as superior, upward social comparison precipitates perceived discrepancy and insecurity. Within fitness social media, this mechanism is amplified by the platforms’ visual centrality and algorithmic curation. Physiques characterized by low adiposity, pronounced muscle definition, and highly polished lifestyles establish unattainable somatic ideals. Evidence indicates that social media appearance comparisons correlate with heightened body dissatisfaction and adverse emotional responses, particularly following repeated exposure to idealized imagery (Fardouly et al., 2015; Ladwig et al., 2024). A longitudinal study by Breves et al. (2025b) further confirms that appearance oriented influencer content sustains elevated appearance comparison among users. Consequently, fitness social media not only disseminates exercise information but also continuously highlights the disparity between users’ actual physiques and aesthetic ideals, thereby generating substantial body image pressure (Breves et al., 2025b).

Complementing social comparison theory, objectification theory (Fredrickson and Roberts, 1997) provides a structural framework for understanding body image pressure. This theory proposes that when sociocultural environments define bodily value through visible aesthetic metrics, individuals internalize this external gaze, habitually evaluating themselves from a third person perspective. McKinley and Hyde (1996) operationalized this phenomenon as body surveillance: a chronic preoccupation with outward presentation rather than internal somatosensory experience or physiological function. In digital fitness contexts, idealized imagery intersects with appearance contingent feedback mechanisms (e.g., likes, comments), predisposing users to treat their bodies as objects requiring continuous display and modification. Recent evidence demonstrates that appearance preoccupation correlates significantly with self-objectification, body shame, and body surveillance, fundamentally degrading somatic appraisal (Brasil et al., 2024). As appearance driven internalized scrutiny permeates actual exercise and physical display contexts, individuals increasingly fear negative evaluation, thereby exacerbating social physique anxiety (Hart et al., 1989; Brasil et al., 2024).

Synthesizing these perspectives, body image pressure fails to translate into sustained exercise adherence. Although perceived discrepancies between actual and ideal physiques may stimulate transient exercise initiation intended for appearance enhancement or evaluation avoidance, such behavior is driven by guilt, shame, and extrinsic pressure rather than intrinsic identification (Teixeira et al., 2012). Consequently, its prolonged sustainability is severely compromised. Longitudinally, Breves et al. (2025a) observed no sustained behavioral gains from exposure to appearance oriented content. Furthermore, conflating exercise with the expectation of observation, comparison, and judgment promotes avoidance behaviors in physical activity settings where evaluative threat is salient. Moffitt (2024) demonstrated that elevated social physique anxiety and negative fitness comparisons correlate with reduced actual exercise participation and a preference for low-threat environments. Ultimately, while body image pressure may prompt initial behavioral engagement, it predominantly erodes intrinsic motivation, enjoyment, and sustained participation, thereby exerting a net negative effect on exercise adherence.

### 2.2 The positive pathway linking fitness social media exposure to exercise adherence

#### 2.2.1 Fitness social media exposure and parasocial relationships

Parasocial relationships denote the unilateral emotional bonds and psychological intimacy audiences develop with media figures through sustained exposure. Foundational research indicates these bonds emerge from cumulative familiarity, perceived accessibility, and imagined interaction, independent of actual reciprocity (Horton and Wohl, 1956; Rubin and McHugh, 1987). Compared to traditional media figures, fitness social media influencers consistently document training regimens, dietary protocols, physiological transformations, and lifestyle details. This continuous self-presentation facilitates the perception of influencers as ubiquitous social actors, engendering companionship, trust, and psychological realism through repeated engagement.

This mechanism is particularly robust within digital fitness communication. Feng et al. (2025) demonstrated that the interactivity, authenticity, and companionship inherent in online fitness services significantly drive parasocial relationship formation. Furthermore, Breves et al. (2025b) established that sustained exposure to fitness oriented content cultivates these unilateral bonds, with effect magnitudes varying by specific content styles. Consequently, increased frequency of fitness social media exposure structurally facilitates the formation of parasocial relationships with influencers. Therefore, the following hypothesis is formulated:

*H1*: Fitness social media exposure positively influences parasocial relationships.

#### 2.2.2 Parasocial relationships and exercise self-efficacy

Parasocial relationships enhance exercise self-efficacy by transforming media figures from generic information providers into relatable behavioral models. Social cognitive theory posits that vicarious experience—observing comparable individuals successfully execute tasks—is a primary source of self-efficacy (Bandura, 2001). Within fitness social media, robust parasocial bonds recontextualize an influencer’s training routines and strategies for overcoming fatigue or time constraints. Rather than perceiving these actions as irrelevant performative content, users interpret them as reproducible, learnable experiences. This psychologically proximal observational learning directly bolsters individuals’ confidence to maintain exercise amidst busyness, fatigue, or emotional distress, aligning with the core conceptualization of exercise self-efficacy (McAuley, 1993).

However, evidence regarding the direct impact of parasocial relationships on exercise outcomes exhibits inconsistencies. For instance, a longitudinal study of health television programming found that parasocial relationships did not reliably enhance self-efficacy or exercise behavior, indicating that such bonds do not inherently translate into actionable outcomes (Siegenthaler et al., 2023). This discrepancy likely stems from the study’s focus on heavily edited television personas, which contrast sharply with digital fitness creators characterized by high interactivity, continuous updates, and daily companionship. Given that authenticity and interactivity facilitate robust parasocial bonds in online environments, and that social media driven parasocial relationships successfully promote health behaviors (Feng et al., 2025; Hoffner and Bond, 2022), these digital bonds systematically make behavioral modeling personally relevant. Consequently, this relevance directly enhances users’ exercise self-efficacy. Therefore, the following hypothesis is formulated:

*H2*: Parasocial relationships positively influence exercise self-efficacy.

#### 2.2.3 Exercise self-efficacy and exercise identity

Exercise self-efficacy denotes the confidence to persist with exercise amidst various barriers, whereas exercise identity represents the assimilation of the “regular exerciser” role into the self-concept. Despite operating at distinct conceptual levels, positive appraisals of physical capability fundamentally underpin the construction of exercise identity. As individuals consistently demonstrate competence in overcoming barriers and sustaining routines, these efficacy judgments are internalized into stable self-perceptions, cementing exercise as a core component of self-definition (Strachan et al., 2026; Rhodes et al., 2025).

While direct empirical validation of the causal link between exercise self-efficacy and identity remains sparse, existing literature offers convergent support. Review evidence indicates that physical activity identity correlates stably with capability appraisals and self-regulatory mechanisms (Rhodes et al., 2016). Furthermore, prospective data demonstrates that perceived behavioral control predicts exercise identity development (De Bruijn et al., 2012). Because perceived behavioral control structurally differs from self-efficacy, this evidence provides indirect rather than definitive support. Consequently, the facilitation of exercise identity by self-efficacy is posited as a theoretical inference necessitating rigorous empirical verification, rather than a fully established empirical fact.

*H3*: Exercise self-efficacy positively influences exercise identity.

#### 2.2.4 Exercise identity and exercise adherence

Exercise identity functions as a pivotal variable in explaining sustained behavioral engagement because it transcends transient behavioral intentions, representing a structural shift in identity definition. Identity theory postulates that individuals align their actions with internalized role meanings to preserve cognitive coherence and identity verification (Burke, 2004). Within physical activity contexts, once the exerciser role is integrated into the core identity, exercise ceases to be an extrinsic temporary regimen; instead, it becomes a routinized practice essential for maintaining psychological consistency. Corroborating this perspective, foundational measurement frameworks conceptualize this construct specifically through the centrality of physical activity to the overall sense of self (Anderson and Cychosz, 1994).

Substantial empirical evidence consistently demonstrates that robust exercise identity structurally facilitates behavioral maintenance. Initial research regarding the consistency between identity and exercise indicated that stronger identity integration predicts higher adherence to corresponding behavioral frequencies (Strachan et al., 2009). Subsequent systematic reviews, randomized controlled trials, and longitudinal studies further establish a stable association between identity strength and subsequent physical activity levels. Moreover, these investigations reveal that identity modifications successfully predict sustained behavioral performance following intervention cessation (Rhodes et al., 2016; Gillman et al., 2021; Porter et al., 2024). Given recent evidence highlighting the critical function of physical activity identity in converting motivation into tangible action (Rhodes et al., 2025), a strengthened identity inherently correlates with elevated adherence. Therefore, the following hypothesis is formulated:

*H4*: Exercise identity positively influences exercise adherence.

### 2.3 The negative pathway linking fitness social media exposure to exercise adherence

#### 2.3.1 Fitness social media exposure and physical appearance comparison

Physical appearance comparison denotes the tendency to evaluate personal morphology, adiposity, muscle definition, and overall aesthetics against external benchmarks. Festinger (1954) postulated that in the absence of objective metrics, individuals rely on social comparison for self-evaluation. Digital platforms continuously provide highly visible, comparable somatic cues. Compared to general lifestyle content, fitness social media disproportionately emphasizes aesthetic training outcomes, adiposity reduction, muscle definition, and dietary results. Consequently, this environment systematically triggers upward somatic comparisons among users. Fardouly et al. (2015) demonstrated that appearance contingent cues within social media significantly elevate appearance comparison and exacerbate body concern. Similarly, Tiggemann and Zaccardo (2015) identified the activation of comparison processes as the primary mechanism through which idealized fitness imagery degrades somatic appraisal.

Within digital fitness contexts, these comparisons transcend general aesthetic judgments, extending to the perceived visibility of training results and the display value of the physique. Robinson et al. (2017) observed that idealized fitness imagery elevates somatic concern without significantly promoting actual exercise behavior. Furthermore, Ladwig et al. (2024) established that appearance oriented social media trends exacerbate body dissatisfaction and negative affect. Recent longitudinal evidence corroborates that appearance driven influencer content sustains elevated appearance comparison among audiences, yet this cognitive shift fails to translate into sustained physical activity (Breves et al., 2025a; Breves et al., 2025b). Therefore, increased exposure to fitness social media inherently strengthens the tendency toward physical appearance comparison. Consequently, the following hypothesis is formulated:

*H5*: Fitness social media exposure positively influences physical appearance comparison.

#### 2.3.2 Physical appearance comparison and body surveillance

While conceptually distinct, physical appearance comparison and body surveillance are deeply interconnected. The former centers on the evaluative process of measuring one’s appearance against others, whereas the latter reflects the habitual tendency to perceive one’s body through the lens of an external observer—prioritizing outward aesthetics over internal bodily sensations (McKinley and Hyde, 1996). According to objectification theory, persistent exposure to an environment where the body is evaluated and scrutinized leads individuals to internalize this external gaze into routine self-monitoring (Fredrickson and Roberts, 1997). Specifically, frequent appearance comparison compels individuals to attend to visible features—such as shape, adiposity, and muscle definition—and prompts habitual verification against external standards. Over time, this comparative process precipitates sustained body surveillance.

Recent empirical evidence from social media contexts underscores this progressive relationship. Seekis et al. (2020) demonstrated that upward appearance comparison, body surveillance, and social appearance anxiety operate sequentially in the development of body image disturbances. Similarly, Bissonette Mink and Szymanski (2022) found that upward comparison and body surveillance serially mediate the link between TikTok usage and body dissatisfaction. Further, Brasil et al. (2024) indicated that preoccupation with social media appearance is strongly associated with self-objectification and surveillance behaviors. Although direct investigations within the fitness-specific social media niche remain sparse, these broader findings suggest a stable psychological trajectory. Users frequently consuming fitness content—often characterized by idealized physiques—are likely to internalize these comparisons, shifting from sporadic evaluation to chronic monitoring of their own appearance. Consequently, this study proposes:

*H6*: Physical appearance comparison positively affects body surveillance.

#### 2.3.3 Body surveillance and social physique anxiety

Social physique anxiety (SPA) is defined as the distress, apprehension, or tension individuals experience when they perceive their bodies to be under the scrutiny or evaluation of others (Hart et al., 1989). While body surveillance involves the cognitive act of monitoring one’s appearance, SPA represents the affective consequence of that monitoring—specifically, a preoccupation with unfavorable external appraisal. Theoretically, habitual body surveillance primes individuals for heightened evaluative sensitivity; by adopting a permanent “outsider’s perspective” on their own physique, individuals increasingly anticipate that others will apply similarly rigorous standards in social interactions. Empirical models support this trajectory: Seekis et al. (2020) demonstrated that body surveillance precipitates social appearance anxiety, and Finn et al. (2024) identified a robust association between surveillance behaviors and the fear of negative evaluation. These findings categorize body surveillance as a critical psychological precursor to evaluative anxiety.

This mechanism is particularly salient in exercise-related contexts. In environments such as gyms or athletic fields, where form-fitting attire is standard, the internalized focus of body surveillance often shifts toward acute concern regarding the visibility of perceived bodily “flaws”. Fitzsimmons-Craft et al. (2012) observed that body surveillance and social comparison exacerbate the psychological burden of SPA, while Brewer et al. (2004) found that elevated SPA correlates with protective self-presentation, such as wearing concealing clothing or avoiding conspicuous spaces. Although direct investigations into this link within the fitness social media domain remain nascent, the existing evidence supports a robust directional inference: among users exposed to idealized fitness content, intensified body surveillance likely heightens susceptibility to social physique anxiety. Consequently, this study proposes:

*H7*: Body surveillance positively affects social physique anxiety.

#### 2.3.4 Social physique anxiety and exercise adherence

Exercise adherence—the consistent maintenance of a physical activity regimen over time—is frequently compromised by social physique anxiety (SPA). Fundamental to SPA is the anticipation of unfavorable evaluation by others (Hart et al., 1989). Within the framework of self-determination theory, Brunet and Sabiston (2009) demonstrated that SPA thwarts the satisfaction of basic psychological needs, thereby disrupting physical activity through maladaptive motivational pathways. Specifically, when exercise environments are perceived as evaluative arenas where the body is “on display,” individuals struggle to cultivate the autonomy and intrinsic motivation necessary for long-term persistence. For users of fitness social media, the tension between idealized digital standards and the fear of real-world scrutiny often recasts exercise as a reactive coping mechanism for appearance pressure rather than a proactive, sustainable health practice.

Crucially, while SPA may occasionally catalyze short-term exercise as a means of appearance management, such externally regulated behaviors lack longitudinal stability. A systematic review by Teixeira et al. (2012) concluded that exercise driven by controlled motivation and external appraisal is significantly less sustainable than that driven by autonomous factors. High levels of SPA are associated with protective self-presentation—including the use of concealing clothing or the avoidance of conspicuous public spaces (Brewer et al., 2004). This avoidance tendency is further corroborated by Moffitt (2024), who observed that individuals who retreat to home-based or secluded exercise settings often report lower self-presentational efficacy and more frequent negative social comparisons. Furthermore, longitudinal evidence suggests that exposure to body-oriented influencer content fails to yield stable increases in physical activity (Breves et al., 2025a). Collectively, SPA appears to erode exercise adherence by reducing psychological comfort in public domains, increasing avoidance behaviors, and undermining autonomous motivation. Consequently, this study proposes:

*H8*: Social physique anxiety negatively affects exercise adherence.

### 2.4 Hypothesized model

**Figure 1:**
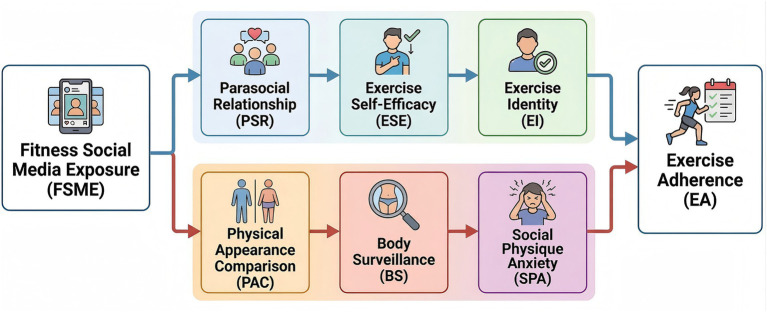
Conceptual framework of the hypothesized model.

Synthesizing the preceding literature review and theoretical frameworks, this study proposes a dual-pathway model to elucidate the relationship between fitness social media exposure and exercise adherence. The model delineates two divergent psychological trajectories. In the facilitative pathway, exposure to fitness content is hypothesized to foster parasocial relationships, which subsequently bolster exercise self-efficacy and solidify exercise identity, ultimately promoting greater exercise adherence. Conversely, in the inhibitory pathway, such exposure may catalyze physical appearance comparison, which intensifies body surveillance and triggers social physique anxiety, thereby undermining exercise adherence. Rather than a monolithic linear effect, this study posits that fitness social media exposure influences behavioral outcomes through distinct, potentially competing psychological mechanisms with asymmetric effects. The comprehensive hypothesized model is illustrated in Figure 1.

## 3 Methods

### 3.1 Instrumentation and scale adaptation

To ensure construct validity and contextual relevance, this study utilized validated psychometric scales, which were refined to align with the specific intersection of fitness social media exposure and sustained exercise behavior. Following the initial drafting, the research team implemented a rigorous multi-stage validation process—comprising source extraction, forward and back-translation, semantic refinement, and pilot testing—to mitigate potential measurement bias stemming from semantic drift.

The measurement items were synthesized and operationalized from several foundational instruments. Fitness social media exposure was assessed using Yang’s (2025) framework, while parasocial relationships were evaluated based on the Rubin and McHugh (1987) classic scale, supplemented by fitness-specific adaptations from Breves et al. (2025b). Exercise self-efficacy was measured following the approaches of McAuley (1993) and Dwyer et al. (1998). For exercise identity, we adapted the Exercise Identity Scale (Anderson and Cychosz, 1994), incorporating recent revisions regarding physical activity identity (Rhodes et al., 2025). Body-related constructs—physical appearance comparison, body surveillance, and social physique anxiety—were quantified using the Physical Appearance Comparison Scale-Revised (Schaefer and Thompson, 2014), the Objectified Body Consciousness Scale (McKinley and Hyde, 1996), and the Social Physique Anxiety Scale (Hart et al., 1989), respectively. Finally, exercise adherence was evaluated via the Exercise Adherence Rating Scale (Newman-Beinart et al., 2017).

Prior to the main data collection, a pilot study (*n* = 53) was conducted to evaluate item clarity, contextual relevance, and initial psychometric properties (Hertzog, 2008). Adhering to established structural equation modeling (SEM) protocols (Wang et al., 2024), we refined the instrument based on item analysis and participant feedback. Specifically, items PAC2 and SPA5 were eliminated due to substantial semantic redundancy and poor discriminant validity. The remaining items underwent minor linguistic adjustments to enhance readability. The finalized questionnaire comprises 33 measurement items (Appendix 1); all item identifiers referenced hereafter correspond to this validated version.

The survey was structured into three distinct modules. First, a screening component filtered participants based on eligibility criteria (e.g., active engagement with fitness-related social media within the preceding month). Second, the core instrument assessed eight latent constructs: fitness social media exposure, parasocial relationships, exercise self-efficacy, exercise identity, physical appearance comparison, body surveillance, social physique anxiety, and exercise adherence. Finally, the third module captured demographic and behavioral data—including gender, age, educational attainment, and preferred platform—to facilitate sample characterization and potential covariate analysis.

All latent variables were operationalized using a seven-point Likert scale, anchored from 1 (“strongly disagree”) to 7 (“strongly agree”), where higher scores signify a greater intensity of the respective construct. This multi-point format was specifically selected to optimize reliability, validity, and discriminant capacity compared to scales with fewer response categories (Dawes, 2008). By leveraging this increased granularity, the study aimed to enhance measurement stability and ensure the data sensitivity required for rigorous structural equation modeling.

### 3.2 Data collection

The proliferation of digital media has established fitness-focused social platforms as primary conduits for health information and exercise guidance (Wang et al., 2026). Beyond mere knowledge dissemination, these platforms exert a subtle yet significant influence on psychological states and the longitudinal commitment to physical activity. Accordingly, this study targeted individuals with recent exposure to fitness-related social media content to examine the associations between digital exposure and exercise adherence, as well as the underlying explanatory mechanisms.

Data were collected between March and April 2026 via an anonymous online survey hosted on the Wenjuanxing platform. The research design strictly adhered to established academic ethical standards. Given the indeterminate size of the target population—comprising national social media users engaged with fitness content—a non-probability sampling design was employed (Gao et al., 2024).

To maximize response rates and capture a diverse cross-section of fitness-content consumers, a three-pronged sampling strategy was implemented. First, convenience sampling was used to distribute the survey through personal networks via instant messaging tools (e.g., WeChat). Second, self-selection sampling was utilized by sharing the survey link within dedicated fitness communities and sections on major platforms, including Weibo and Xiaohongshu. Finally, a snowball sampling technique was employed, wherein initial respondents recruited peers who maintained regular exercise habits or frequently engaged with fitness media (Ivanov et al., 2024; Lin and Yu, 2025; Xia et al., 2024).

Of the 726 total responses collected, 86 were excluded via the initial screening module as these respondents had not engaged with fitness-related social media content in the preceding month. A further 65 cases were discarded following a rigorous data-quality audit, which identified invalid entries characterized by insufficient completion times or invariant response patterns (e.g., straight-lining). This resulted in a final analytical sample of 575 valid questionnaires.

The adequacy of the sample size was evaluated against established requirements for partial least squares structural equation modeling (PLS-SEM). Power in PLS-SEM is typically determined by model complexity, specifically the maximum number of structural paths directed at any single endogenous construct (Hair et al., 2017; Lin et al., 2023). Given the moderate structural complexity of the hypothesized model, the final sample of 575 substantially exceeds recommended heuristic thresholds, ensuring robust parameter estimation. Furthermore, this sample size provides ample empirical power for fuzzy-set qualitative comparative analysis (fsQCA) involving seven antecedent conditions and one outcome, facilitating a rigorous assessment of both necessary and sufficient configurations. The demographic profile of the participants is summarized in Table 1.

**Table 1:** Respondent demographics (*n* = 575).

Variable	Category	Frequency	%
Gender	Male	417	72.5
	Female	158	27.5
Age	18–22 years	380	66.1
	23–27 years	122	21.2
	28–32 years	43	7.5
	33–40 years	21	3.7
	41 years and above	9	1.6
Highest education	Junior college	41	7.1
	Bachelor’s degree	414	72.0
	Master’s degree	92	16.0
	Doctoral degree or above	28	4.9
Primary platform used	Xiaohongshu	72	12.5
	Weibo	18	3.1
	Douyin	403	70.1
	Bilibili	46	8.0
	WeChat Channels	20	3.5
	Others	16	2.8

### 3.3 Data analysis

We employed a dual-methodological approach for data analysis, combining partial least squares structural equation modeling (PLS-SEM) and fuzzy-set qualitative comparative analysis (fsQCA). PLS-SEM, executed via SmartPLS 4, was utilized for hypothesis testing. We selected this method for its robustness in handling complex models with multiple latent variables and path relationships, enabling a direct comparison of the relative strengths of positive and negative pathways. Consequently, our analysis prioritized the identification of key paths and the explanatory power of endogenous variables over mere model fit (Dash and Paul, 2021; Hair et al., 2019). To complement this net-effect paradigm, we incorporated fsQCA to elucidate the combinatorial conditions driving high exercise adherence. By accommodating causal asymmetry and equifinality—where disparate condition configurations yield identical outcomes—fsQCA provides vital configurational insights that synergize seamlessly with the net-effect estimations of PLS-SEM (Ragin, 2008; Fiss, 2011; Pappas and Woodside, 2021).

In accordance with established guidelines (Hair et al., 2019), our PLS-SEM procedure entailed sequential assessments of the measurement and structural models. The measurement model evaluation verified the internal consistency reliability and convergent validity of each latent variable using item outer loadings, Cronbach’s alpha, composite reliability, and average variance extracted (AVE) (Hair et al., 2017; Hair et al., 2019; Yu et al., 2023). Discriminant validity was established via the Fornell-Larcker criterion and the heterotrait-monotrait ratio (HTMT) (Fornell and Larcker, 1981; Henseler et al., 2015). For the structural model, path significance was determined using 5,000 bootstrap resamples, with a specific focus on evaluating the relative explanatory power of contrasting pathways (Hair et al., 2019). Subsequent to the PLS-SEM evaluation, fsQCA was applied to capture the non-linear, synergistic effects of multiple antecedents. We conducted necessity analyses, sufficiency configuration analyses, and robustness checks to delineate the specific condition configurations that engender both high and non-high exercise adherence, thereby revealing the complex interactions shaping the ultimate outcome (Ragin, 2008; Fiss, 2011; Pappas and Woodside, 2021).

## 4 Results

### 4.1 Symmetric analysis

#### 4.1.1 Measurement model assessment

We utilized partial least squares structural equation modeling (PLS-SEM), executed via SmartPLS 4, to test the research hypotheses. Prior to structural evaluation, we assessed the measurement model to establish the reliability and validity of the latent constructs. Internal consistency reliability and convergent validity were verified using item outer loadings, Cronbach’s alpha, composite reliability (CR), and average variance extracted (AVE). As detailed in Table 2, all outer loadings exceeded 0.40, with the majority surpassing the 0.70 threshold, demonstrating satisfactory item-construct associations and overall loading robustness (Hair et al., 2017; Hair et al., 2019). Although the loading for item SPA2 was marginal (0.604), it was retained to preserve the construct’s content validity, given that the CR (0.850) and AVE (0.591) for social physique anxiety remained well above acceptable thresholds.

**Table 2:** Construct reliability and validity results.

Constructs	Items	Loadings	Cronbach’s Alpha	Composite reliability	Average variance extracted (AVE)
BS	BS1	0.823	0.855	0.900	0.692
	BS2	0.865			
	BS3	0.853			
	BS4	0.785			
EA	EA1	0.889	0.905	0.934	0.779
	EA2	0.898			
	EA3	0.877			
	EA4	0.867			
EI	EI1	0.853	0.847	0.897	0.686
	EI2	0.856			
	EI3	0.804			
	EI4	0.797			
ESE	ESE1	0.925	0.913	0.935	0.743
	ESE2	0.857			
	ESE3	0.838			
	ESE4	0.842			
	ESE5	0.845			
FSME	FSME1	0.83	0.845	0.895	0.682
	FSME2	0.84			
	FSME3	0.779			
	FSME4	0.853			
PAC	PAC1	0.781	0.767	0.863	0.678
	PAC2	0.900			
	PAC3	0.783			
PSR	PSR1	0.842	0.885	0.916	0.686
	PSR2	0.806			
	PSR3	0.845			
	PSR4	0.813			
	PSR5	0.833			
SPA	SPA1	0.792	0.775	0.850	0.591
	SPA2	0.604			
	SPA3	0.862			
	SPA4	0.793			

We rigorously established discriminant validity using both the Fornell-Larcker criterion and the heterotrait-monotrait (HTMT) ratio. As Table 3 illustrates, the square root of the AVE for each construct exceeded its bivariate correlations with all other constructs, fulfilling the Fornell-Larcker conditions (Fornell and Larcker, 1981; Li et al., 2025). Concurrently, all HTMT ratios fell below the conservative 0.85 threshold, confirming explicit differentiation among the latent variables (Henseler et al., 2015). Furthermore, given our reliance on a unified self-report instrument, we screened for common method bias (CMB) via full-collinearity variance inflation factors (VIFs) and Harman’s single-factor test. All VIF values remained below 5, and the primary extracted factor accounted for less than 50% of the total variance, indicating that CMB did not significantly confound our dataset (Kock, 2015; Podsakoff et al., 2003). Consequently, the measurement model exhibited the requisite robustness for subsequent structural evaluation.

**Table 3:** Fornell-Larcker test and heterotrait-monotrait ratio (HTMT) test results.

Constructs	BS	EA	EI	ESE	FSME	PAC	PSR	SPA
Fornell-Larcker test results								
BS	0.832							
EA	0.035	0.883						
EI	0.036	0.628	0.828					
ESE	0.060	0.459	0.682	0.862				
FSME	0.062	0.107	0.161	0.216	0.826			
PAC	0.104	0.051	0.079	0.033	0.091	0.823		
PSR	0.094	0.129	0.273	0.368	0.474	0.035	0.828	
SPA	0.067	−0.092	−0.027	−0.013	0.033	0.008	0.012	0.769
Heterotrait-monotrait ratio (HTMT) test results								
BS								
EA	0.050							
EI	0.048	0.716						
ESE	0.068	0.503	0.772					
FSME	0.070	0.121	0.189	0.242				
PAC	0.117	0.067	0.104	0.051	0.113			
PSR	0.102	0.144	0.316	0.408	0.541	0.059		
SPA	0.085	0.106	0.057	0.049	0.076	0.059	0.059	

#### 4.1.2 Structural model assessment

**Figure 2:**
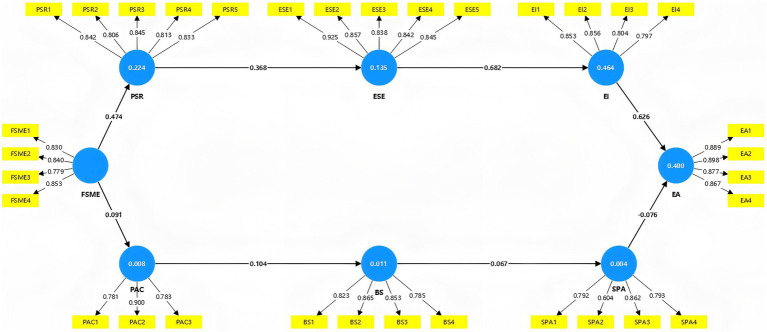
Structural model path coefficients and R^2^ values.

We estimated the structural model using the PLS-SEM algorithm in SmartPLS 4, applying a path-weighting scheme to obtain standardized results (Hair et al., 2017, 2019). Statistical significance was determined via nonparametric bootstrapping (5,000 resamples, two-tailed, *α* = 0.05) (Hair et al., 2017, 2019). To compare the relative explanatory power of the positive and negative pathways, we evaluated the structural model based on standardized path coefficients (*β*), t-values, *p*-values, and coefficients of determination (R^2^) (Figure 2; Table 4).

**Table 4:** Results of main model.

Path type	Hypotheses	Paths	Path coefficient	T values	*p* values	Decision
Positive path	H1	FSME → PSR	0.474	14.332	<0.001	Supported
	H2	PSR → ESE	0.368	9.467	<0.001	Supported
	H3	ESE → EI	0.682	27.959	<0.001	Supported
	H4	EI → EA	0.626	19.954	<0.001	Supported
Negative path	H5	FSME → PAC	0.091	2.094	0.036	Supported
	H6	PAC → BS	0.104	2.041	0.041	Supported
	H7	BS → SPA	0.067	1.211	0.226	Not supported
	H8	SPA → EA	−0.076	1.994	0.046	Supported

Analysis of the positive pathway revealed a cascading sequence of significant, positive effects. Fitness social media exposure strongly predicted parasocial relationships (β = 0.474, t = 14.332, *p* < 0.001), supporting H1. In turn, these parasocial relationships enhanced exercise self-efficacy (β = 0.368, t = 9.467, *p* < 0.001), which subsequently drove exercise identity (β = 0.682, t = 27.959, *p* < 0.001) and ultimate exercise adherence (β = 0.626, t = 19.954, *p* < 0.001), thereby supporting H2, H3, and H4, respectively. Furthermore, the R^2^ values for these constructs (parasocial relationships: 0.224; self-efficacy: 0.135; identity: 0.464; adherence: 0.400) indicate that the pathway’s explanatory power for exercise adherence is predominantly concentrated in the proximal stages of identity and self-efficacy, rather than in more distal antecedents (Figure 2).

Conversely, the negative pathway exhibited weaker and partially disrupted relationships. Fitness social media exposure positively predicted physical appearance comparison (β = 0.091, t = 2.094, *p* = 0.036), which in turn increased body surveillance (β = 0.104, t = 2.041, *p* = 0.041), supporting H5 and H6. However, this cascading effect was interrupted, as body surveillance failed to significantly predict social physique anxiety (β = 0.067, t = 1.211, *p* = 0.226), leading to the rejection of H7. Despite this break, social physique anxiety itself exerted a significant negative impact on exercise adherence (β = −0.076, t = 1.994, *p* = 0.046), supporting H8. Furthermore, the variance explained (R^2^) by the constructs in this negative pathway was negligible (physical appearance comparison: 0.008; body surveillance: 0.011; social physique anxiety: 0.004). Ultimately, these findings reveal an asymmetrical structural model (Figure 2): the association between fitness social media and exercise adherence is driven predominantly by positive psychological mechanisms, whereas the negative pathway offers only marginal and fragmented explanatory value.

### 4.2 Asymmetric analysis

#### 4.2.1 Calibration

A fundamental preparatory step in fuzzy-set qualitative comparative analysis (fsQCA) is data calibration, which maps raw continuous scores onto set membership values ranging from 0 to 1 (Table 5) (Ragin, 2009). Applying the direct calibration method, we established anchors at the 0.95, 0.50, and 0.05 quantiles for each variable to denote full membership, the crossover point, and full non-membership, respectively (e.g., for a 7-point scale, full membership corresponded to approximately 6.2, the crossover point to 4.0, and full non-membership to 1.8) (Chen et al., 2025; Ragin, 2014; Wang et al., 2025a). We opted for a data-driven, quantile-based anchoring approach rather than external theoretical thresholds because the contextually adapted seven-point scales used in this study lack unified substantive benchmarks across constructs. In this context, quantile calibration provides a more robust mechanism for systematically identifying relatively high and low states within the sample.

**Table 5:** Descriptive statistics of calibrated fuzzy-set membership scores.

Constructs	Mean (SD)	Min	Max	*N*
Fitness social media exposure (FSME)	0.51 (0.30)	0.01	0.95	575
Parasocial relationship (PSR)	0.52 (0.31)	0.01	0.96	575
Exercise self-efficacy (ESE)	0.52 (0.30)	0.01	0.95	575
Exercise identity (EI)	0.51 (0.30)	0.01	0.95	575
Physical appearance comparison (PAC)	0.50 (0.33)	0.00	0.98	575
Body surveillance (BS)	0.53 (0.30)	0.00	0.95	575
Social physique anxiety (SPA)	0.48 (0.30)	0.01	0.97	575
Exercise adherence (EA)	0.52 (0.30)	0.02	0.97	575

#### 4.2.2 Analysis of necessity

Following established fsQCA conventions, a condition must exhibit a consistency score of 0.90 or higher to be deemed necessary (Fiss, 2011; Greckhamer et al., 2018; Wang et al., 2025b; Yu et al., 2025). Our analysis revealed that no single antecedent serves as a necessary prerequisite for either high or non-high exercise adherence (Table 6). For the outcome of high exercise adherence, all individual conditions yielded consistency values below this threshold; exercise identity (EI) and exercise self-efficacy (ESE) demonstrated the highest consistencies (0.791 and 0.754, respectively), with corresponding coverages of 0.807 and 0.754. Similarly, for non-high exercise adherence, the absence of exercise identity (~EI) and the absence of exercise self-efficacy (~ESE) emerged as the strongest conditions, yet their consistencies (0.798 and 0.738) and coverages (0.783 and 0.739) still fell short of the 0.90 benchmark. Consequently, while EI and ESE represent critical variables within the sample, neither independently dictates the outcome. Instead, they must configure with other media- or body-image-related factors to drive specific states of exercise adherence.

**Table 6:** Analysis of necessary conditions for high and low exercise adherence.

Causal conditions	Exercise adherence		~Exercise adherence	
	Consistency	Coverage	Consistency	Coverage
FSME	0.684	0.687	0.649	0.612
~FSME	0.614	0.650	0.669	0.666
PSR	0.692	0.689	0.633	0.593
~PSR	0.591	0.632	0.668	0.670
ESE	0.754	0.754	0.585	0.550
~ESE	0.550	0.585	0.738	0.739
EI	0.791	0.807	0.558	0.535
~EI	0.544	0.567	0.798	0.783
PAC	0.649	0.667	0.628	0.606
~PAC	0.617	0.638	0.655	0.637
BS	0.690	0.675	0.677	0.623
~BS	0.615	0.670	0.647	0.662
SPA	0.623	0.664	0.658	0.659
~SPA	0.681	0.679	0.665	0.624

#### 4.2.3 Analysis of sufficiency

Guided by established fsQCA protocols and our sample characteristics, we constructed the truth table applying strict thresholds: a consistency of 0.80, a case frequency of 5, and a proportional reduction in inconsistency (PRI) of 0.70 (Foroughi et al., 2024). Whereas the preceding PLS-SEM analysis evaluated symmetric net effects across the sample, fsQCA captures the complex, asymmetric equifinality of these pathways. We report the intermediate solution as our primary framework, cross-referencing it with the parsimonious solution to differentiate core from peripheral conditions. All identified configurations yielded high consistency values exceeding 0.90 (Table 7). Collectively, the overall solution coverage (0.62) and consistency (0.89) confirm that these configurational paths robustly explain the emergence of high exercise adherence.

**Table 7:** Configurations for high and low exercise adherence.

Types	High level of EA								Low level of EA
Causal conditions	C1	C2	C3	C4	C5	C6	C7	C8	C9
FSME			•			•	⨂	⨂	⨂
PSR	•		•	•	⊗	⊗	⨂		⊗
ESE	●	●	●	●	●	●	⨂	●	⊗
EI	●	●	●	●	●	●	●	●	⨂
PAC	⨂	⨂	●	●	●		●	⨂	⨂
BS		•		●	⨂	•	●		⨂
SPA	⊗	⊗			⊗	•	⨂	⊗	⨂
Raw coverage	0.33	0.32	0.38	0.36	0.25	0.26	0.22	0.31	0.26
Unique coverage	0.01	0.01	0.03	0.01	0.01	0.02	0.02	0.01	0.26
Consistency	0.93	0.93	0.91	0.92	0.95	0.95	0.95	0.93	0.92
Overall coverage	0.62								0.26
Overall consistency	0.89								0.92

Solid black circles indicate the presence of a condition, whereas circled crosses indicate the absence or negation of a condition. Large symbols denote core conditions, and small symbols denote peripheral conditions. Blank cells indicate irrelevant or “don’t care” conditions.

#### 4.2.4 Robustness checks

To rigorously verify the stability of our configurational findings, we conducted robustness checks. In accordance with established fsQCA guidelines, we reanalyzed the truth table after increasing the consistency threshold to 0.85 (Greckhamer et al., 2018; Pappas and Woodside, 2021). The results demonstrated that the core combinations of conditions for both high and non-high exercise adherence remained qualitatively consistent, with only marginal variations in overall consistency and coverage. These findings confirm that the identified configurations are not artifacts of specific parameter settings, but rather exhibit strong robustness and replicability. Ultimately, this reinforces that the explanatory core for high exercise adherence is threshold-independent, remaining stably anchored in exercise self-efficacy and exercise identity.

## 5 Discussion and implications

### 5.1 Discussion of the SEM results

Our SEM analysis reveals that the relationship between fitness social media exposure and exercise adherence is multidimensional, comprising both facilitative and risk mechanisms with markedly asymmetrical explanatory power. Specifically, fitness social media exposure robustly promoted parasocial relationships, whereas its influence on physical appearance comparison, though significant, was substantially weaker. Consequently, the variance explained in exercise adherence was driven predominantly by the positive pathway, with the negative pathway offering limited predictive value. Given this pronounced discrepancy and the substantial R^2^ value for exercise adherence, we infer that our sample primarily experienced fitness social media as a relational and motivational health communication environment, rather than merely a catalyst for appearance-based discipline. This aligns with a recent systematic review (Powell and Pring, 2024) indicating that while health influencers can generate both beneficial and harmful outcomes, detrimental effects are mostly localized to body image concerns. Synthesizing these perspectives, we propose a nuanced conclusion: fitness social media is not unequivocally beneficial; rather, its positive functions were readily activated within our cohort, whereas its detrimental effects appeared highly conditional (Hoffner and Bond, 2022; Yang, 2025).

Specifically, the sequence of parasocial relationships, exercise self-efficacy, and exercise identity emerged as the most robust and explanatory mediational chain in our model. Within this pathway, self-efficacy exerted the strongest influence on exercise identity, which subsequently drove exercise adherence. These dynamics suggest that translating fitness social media engagement into sustained behavioral change relies not on fleeting emotional stimulation, but on cultivating durable self-regulatory resources. Ultimately, users must develop the confidence to maintain routine physical activity and internalize exercise into their core self-concept. This conclusion corroborates social cognitive theory, which posits self-efficacy as central to behavioral maintenance (Bandura, 2001), and aligns with recent evidence highlighting the role of physical activity identity in fostering long-term adherence (Gillman et al., 2021; Porter et al., 2024; Rhodes et al., 2025).

Analysis of the negative pathway indicates that body image pressures do not inevitably propagate linearly. Although fitness social media exposure precipitated physical appearance comparison—which subsequently heightened body surveillance—this surveillance failed to significantly translate into social physique anxiety. The non-significant link from body surveillance to social physique anxiety may reflect the specific fitness context or sample characteristics. Nevertheless, when social physique anxiety did manifest, it significantly undermined exercise adherence. This decoupling highlights critical contextual and individual boundary conditions that moderate the transition from perceiving body discrepancies to fearing social evaluation. For instance, variables such as platform style, gender composition, the degree of physical exposure in actual exercise settings, and preexisting body evaluation paradigms likely dictate whether this detrimental chain is fully activated. This aligns with recent literature demonstrating that appearance preoccupation, self-objectification, and social evaluation concerns rarely unfold in a strict linear sequence; rather, they are contingent upon specific digital environments and individual psychological profiles (Brasil et al., 2024; Finn et al., 2024; Moffitt, 2024).

### 5.2 Discussion of the QCA results

The fsQCA results reveal that no single condition serves as a necessary prerequisite for high exercise adherence, offering a critical asymmetric complement to the symmetric net-effect conclusions derived from our SEM analysis. Despite exhibiting the highest consistency metrics, neither the presence of exercise identity (for high adherence) nor its absence (for non-high adherence) independently met the necessity threshold. Consequently, the emergence of sustained exercise behavior is not dictated by any isolated antecedent, but rather by the synergistic interplay of multiple conditions. In this context, while exercise identity and self-efficacy remain highly salient across most high-adherence configurations, their effects operate configurationally rather than independently. This finding fundamentally aligns with the core epistemological premise of fsQCA: complex behavioral outcomes are generated by configurational equifinality rather than the independent operation of single variables (Fiss, 2011; Greckhamer et al., 2018).

Crucially, the emergence of high exercise adherence demonstrated pronounced equifinality. Based on the configurational solutions (Table 7), we typify these pathways into four distinct archetypes. The first is an intrinsic-resource-dominant pathway (Configurations C1, C2, and C8), which indicates that high exercise adherence can emerge when exercise self-efficacy and exercise identity are jointly present, even when media-related or appearance-related conditions are absent, peripheral, or irrelevant. The second is a media-relationship-reinforcement pathway (C3 and C4). Here, fitness social media exposure and/or parasocial relationships coexist with self-efficacy and identity, suggesting that media and relational cues can further consolidate sustained exercise behavior; notably, appearance-related conditions in these configurations do not necessarily obstruct adherence. The third is a self-regulatory-compensation pathway (C5 and C6). In these configurations, high adherence is maintained despite the absence of parasocial relationships and, in C6, the absence of fitness social media exposure; this suggests that exercise self-efficacy and exercise identity can compensate for weak media-relational support. The fourth is an identity-based appearance-engagement pathway (C7), where exercise identity, physical appearance comparison, and body surveillance are present, whereas fitness social media exposure, parasocial relationships, exercise self-efficacy, and social physique anxiety are absent. This configuration should not be interpreted as evidence that self-efficacy is universally required; rather, it suggests that a strong exercise identity may sustain adherence when appearance-related engagement coexists with the absence of social physique anxiety.

Furthermore, the low-adherence configuration occupied a distinct explanatory space, indicating that high and low adherence are governed by different generative logics rather than being mere symmetrical inversions of a single paradigm. As corroborated by our robustness checks, these core configurations remained fundamentally stable across elevated consistency thresholds, confirming they are not mere artifacts of parameter selection. Methodologically, relying exclusively on SEM would have captured significant net effects across the aggregate sample, yet obscured the equifinality by which varying configurations yield identical outcomes. Conversely, fsQCA highlights that the explanatory core of sustained adherence is not fitness social media exposure per se, but rather proximal self-regulatory and identity-related resources, with exercise identity appearing particularly stable across high-adherence configurations. Ultimately, this synthesis confirms that PLS-SEM and fsQCA are complementary rather than interchangeable; together, they decode the complexity of causal mechanisms through integrated symmetric and asymmetric perspectives (Greckhamer et al., 2018; Pappas and Woodside, 2021).

### 5.3 Theoretical implications

Theoretically, this study makes three salient contributions. First, it transcends the traditional binary discourse regarding the benefits and detriments of fitness social media. By articulating a comprehensive dual-pathway framework, we establish that positive, facilitative mechanisms possess vastly superior explanatory power over negative risk pathways in driving exercise adherence. Second, our findings elucidate that the stable translation of digital communication into sustained behavior relies not on mere media exposure, but on the mobilization of proximal psychological resources—namely, parasocial relationships, self-efficacy, and exercise identity. Third, by synergizing symmetric net-effect and asymmetric configurational analyses, we demonstrate that traditionally maladaptive pressures, such as physical appearance comparison, do not inevitably degrade exercise adherence. Within specific configurations, robust self-regulatory resources can neutralize these threats, offering a highly granular and nuanced understanding of the complex consequences of digital fitness communication. Collectively, these insights corroborate the paradoxical impact of health influencers while extending the theoretical boundaries of parasocial relationships and physical activity identity within contemporary digital health contexts (Hoffner and Bond, 2022; Powell and Pring, 2024; Rhodes et al., 2025).

Methodologically, integrating PLS-SEM and fsQCA yields substantial analytical synergy. While PLS-SEM captured the symmetric net effects and dominant mediational pathways—establishing baseline statistical robustness across the aggregate sample—fsQCA illuminated the causal asymmetry and equifinality underlying these relationships. Specifically, it demonstrated that high and non-high exercise adherence are governed by distinct, multifaceted configurations rather than a singular linear logic. Consequently, this methodological synthesis transcends mere triangulation; it juxtaposes macro-level linear relationships against nuanced, configurational causal mechanisms. This dual approach fundamentally aligns with advanced scholarly paradigms advocating the investigation of complex causality and equifinality (Fiss, 2011; Pappas and Woodside, 2021).

Practically, to genuinely foster long-term exercise adherence, digital platforms and fitness content creators must pivot their design strategies. Content architectures should prioritize camaraderie, actionable guidance, and transparent progress tracking, rather than perpetuating hyper-idealized physique standards. As Dennehy et al. (2024) highlighted in a recent systematic review, productive engagement with fitness media extends beyond passive viewing to encompass active interaction and self-management; conversely, the relentless barrage of idealized imagery exacerbates psychological risks. Building on this, our findings emphasize that the behavioral impact of fitness social media is not contingent merely on the volume of exposure. Rather, its true efficacy hinges on the content’s capacity to cultivate robust exercise self-efficacy and a resilient physical activity identity—the critical psychosocial resources essential for driving sustained exercise adherence.

For public health practitioners, physical educators, and general users, our findings indicate that influencer-driven interventions must transcend simple metrics of exposure volume or account popularity. Rather, these initiatives should actively cultivate exercise self-efficacy and identity while mitigating the risk of social evaluative threats. For vulnerable demographics—particularly those prone to social physique anxiety and maladaptive comparison—content curation should prioritize functional training, bodily capabilities, and incremental progress over hyper-idealized physical displays. Ultimately, digital fitness communication designed for sustained behavioral change must abandon discrepancy-driven, anxiety-inducing paradigms. Instead, it must champion supportive relational dynamics, enhanced perceived competence, and the consolidation of a resilient exercise identity (Moffitt, 2024; Yang, 2025).

## 6 Limitations and future directions

These findings must be interpreted in light of several limitations. First, because our conclusions are derived from a specific sample and research context, their generalizability is inherently restricted. They are best conceptualized as contextually bound empirical insights, necessitating further validation across more diverse demographic and cultural settings. Second, constrained by our target population and measurement paradigms, the structural model remains inherently parsimonious; it does not exhaust the myriad psychosocial mechanisms and boundary conditions governing the digital fitness communication–exercise adherence nexus. Third, while the integration of PLS-SEM and fsQCA provides robust analytical synergy, these configurational findings require triangulation through alternative analytical frameworks. Due to the cross-sectional design, causal relationships cannot be inferred from the present findings. Moving forward, future research should leverage longitudinal datasets, granular subgroup stratifications, and cross-cultural contexts to interrogate the robustness of these pathways. Specifically, elucidating how algorithmic platform characteristics, individual psychological typologies, and broader sociocultural paradigms moderate these behavioral outcomes will be paramount for advancing the field of digital health communication.

## Data Availability

The raw data supporting the conclusions of this article will be made available by the authors, without undue reservation.

## References

[ref1] Anderson D. F. Cychosz C. M. (1994). Development of an exercise identity scale. Percept. Mot. Skills 78, 747–751. doi: 10.1177/003151259407800313, 8084685

[ref2] André N. Grousset M. Audiffren M. (2024). A behavioral perspective for improving exercise adherence. Sports Med. 10:56. doi: 10.1186/s40798-024-00714-8, 38763991 PMC11102891

[ref3] Bandura A. (2001). Social cognitive theory: an agentic perspective. Annu. Rev. Psychol. 52, 1–26. doi: 10.1146/annurev.psych.52.1.1, 11148297

[ref4] Bissonette Mink D. Szymanski D. M. (2022). TikTok use and body dissatisfaction: examining direct, indirect, and moderated relations. Body Image 43, 205–216. doi: 10.1016/j.bodyim.2022.09.006, 36191378

[ref5] Brasil K. M. Mims C. E. Pritchard M. E. Mcdermott R. C. (2024). Social media and body image: relationships between social media appearance preoccupation, self-objectification, and body image. Body Image 51:101767. doi: 10.1016/j.bodyim.2024.101767, 39018644

[ref6] Breves P. L. Boerman S. C. Stein J. P. Ischen C. van Berlo Z. M. (2025b). The impact of body-positive and fitspirational influencers on body satisfaction: a longitudinal study of evolving parasocial relationships. Hum. Commun. Res. 52, 90–105. doi: 10.1093/hcr/hqaf029

[ref7] Breves P. L. van Berlo Z. M. Teunissen L. König L. Binder A. Naderer B. (2025a). Happier and healthier? Investigating the longitudinal impact of body-positive and fitspirational influencers on weight satisfaction, healthy eating, and physical activity. Health Commun. 40, 2522–2534. doi: 10.1080/10410236.2025.2465795, 39996327

[ref8] Brewer B. W. Diehl N. S. Cornelius A. E. Joshua M. D. Van Raalte J. L. (2004). Exercising caution: social physique anxiety and protective self-presentational behaviour. J. Sci. Med. Sport 7, 47–55. doi: 10.1016/S1440-2440(04)80043-4, 15139164

[ref9] Brunet J. Sabiston C. M. (2009). Social physique anxiety and physical activity: a self-determination theory perspective. Psychol. Sport Exerc. 10, 329–335. doi: 10.1016/j.psychsport.2008.11.002

[ref10] Burke P. J. (2004). Identities and social structure: the 2003 Cooley-Mead award address. Soc. Psychol. Q. 67, 5–15. doi: 10.1177/019027250406700103

[ref11] Chen X. Yu T. Dai J. Jing Y. Wang C. (2025). Unveiling learners’ intentions toward influencer-led education: an integration of qualitative and quantitative analysis. Interact. Learn. Environ. 33, 3469–3487. doi: 10.1080/10494820.2024.2444533

[ref12] Dash G. Paul J. (2021). CB-SEM vs PLS-SEM methods for research in social sciences and technology forecasting. Technol. Forecast. Soc. Change 173:121092. doi: 10.1016/j.techfore.2021.121092

[ref13] Dawes J. (2008). Do data characteristics change according to the number of scale points used? An experiment using 5-point, 7-point and 10-point scales. Int. J. Mark. Res. 50, 61–104. doi: 10.1177/147078530805000106

[ref14] de Bruijn G. J. Verkooijen K. de Vries N. K. van den Putte B. (2012). Antecedents of self identity and consequences for action control: an application of the theory of planned behaviour in the exercise domain. Psychol. Sport Exerc. 13, 771–778. doi: 10.1016/j.psychsport.2012.05.008

[ref15] Dennehy D. P. Murphy S. Foley S. McCarthy J. Morrissey K. (2024). Keeping fit & staying safe: a systematic review of women’s use of social media for fitness. Int. J. Hum. Comput. Stud. 192:103361. doi: 10.1016/j.ijhcs.2024.103361

[ref16] Dwyer J. J. M. Allison K. R. Makin S. (1998). Internal structure of a measure of self-efficacy in physical activity among high school students. Soc. Sci. Med. 46, 1175–1182. doi: 10.1016/S0277-9536(97)10045-4, 9572607

[ref17] Fardouly J. Diedrichs P. C. Vartanian L. R. Halliwell E. (2015). Social comparisons on social media: the impact of Facebook on young women’s body image concerns and mood. Body Image 13, 38–45. doi: 10.1016/j.bodyim.2014.12.002, 25615425

[ref18] Feng Y. Meng J. Cheah J.-H. (2025). From virtual trainers to companions? Examining how digital agency types, anthropomorphism, and support shape Para-social relationships in online fitness. Psychol. Mark. 42, 842–865. doi: 10.1002/mar.22154

[ref19] Festinger L. (1954). A theory of social comparison processes. Hum. Relations 7, 117–140. doi: 10.1177/001872675400700202

[ref20] Finn D. Cardini F. Aspell J. E. Swami V. Todd J. (2024). The impact of body image on social cognition: fear of negative evaluation mediates the relationship between body surveillance and interpersonal distance in women. Body Image 51:101777. doi: 10.1016/j.bodyim.2024.101777, 39128330

[ref21] Fiss P. C. (2011). Building better causal theories: a fuzzy set approach to typologies in organization research. Acad. Manag. J. 54, 393–420. doi: 10.5465/amj.2011.60263120

[ref22] Fitzsimmons-Craft E. E. Harney M. B. Brownstone L. M. Higgins M. K. Bardone-Cone A. M. (2012). Examining social physique anxiety and disordered eating in college women: the roles of social comparison and body surveillance. Appetite 59, 796–805. doi: 10.1016/j.appet.2012.08.019, 22925847

[ref23] Fornell C. Larcker D. F. (1981). Evaluating structural equation models with unobservable variables and measurement error. J. Mark. Res. 18, 39–50. doi: 10.1177/002224378101800104

[ref24] Foroughi B. Senali M. G. Iranmanesh M. Khanfar A. Ghobakhloo M. Annamalai N. . (2024). Determinants of intention to use ChatGPT for educational purposes: findings from PLS-SEM and fsQCA. Int. J. Hum. Comput. Interact. 40, 4501–4520. doi: 10.1080/10447318.2023.2226495

[ref25] Fredrickson B. L. Roberts T.-A. (1997). Objectification theory: toward understanding women’s lived experiences and mental health risks. Psychol. Women Q. 21, 173–206. doi: 10.1111/j.1471-6402.1997.tb00108.x

[ref26] Gao Z. Cheah J. H. Lim X. J. Luo X. (2024). Enhancing academic performance of business students using generative AI: an interactive-constructive-active-passive (ICAP) self-determination perspective. Int. J. Manag. Educ. 22:100958. doi: 10.1016/j.ijme.2024.100958

[ref27] Gillman A. S. Stevens C. J. Bryan A. D. (2021). Women's exercise identity increases after a 16-week exercise RCT and is linked to behavior maintenance at follow-up. Psychol. Sport Exerc. 54:101888. doi: 10.1016/j.psychsport.2021.101888, 33633498 PMC7901813

[ref28] Greckhamer T. Furnari S. Fiss P. C. Aguilera R. V. (2018). Studying configurations with qualitative comparative analysis: best practices in strategy and organization research. Strateg. Organ. 16, 482–495. doi: 10.1177/1476127018786487

[ref29] Hair J. F. Hollingsworth C. L. Randolph A. B. Chong A. Y. L. (2017). An updated and expanded assessment of PLS-SEM in information systems research. Ind. Manag. Data Syst. 117, 442–458. doi: 10.1108/IMDS-04-2016-0130

[ref30] Hair J. F. Risher J. J. Sarstedt M. Ringle C. M. (2019). When to use and how to report the results of PLS-SEM. Eur. Bus. Rev. 31, 2–24. doi: 10.1108/EBR-11-2018-0203

[ref31] Hart E. A. Leary M. R. Rejeski W. J. (1989). Tie measurement of social physique anxiety. J. Sport Exerc. Psychol. 11, 94–104. doi: 10.1123/jsep.11.1.94

[ref32] Henseler J. Ringle C. M. Sarstedt M. (2015). A new criterion for assessing discriminant validity in variance-based structural equation modeling. J. Acad. Mark. Sci. 43, 115–135. doi: 10.1007/s11747-014-0403-8

[ref33] Hertzog M. A. (2008). Considerations in determining sample size for pilot studies. Res. Nurs. Health 31, 180–191. doi: 10.1002/nur.20247, 18183564

[ref34] Hoffner C. A. Bond B. J. (2022). Parasocial relationships, social media, and well-being. Curr. Opin. Psychol. 45:101306. doi: 10.1016/j.copsyc.2022.101306 35219157

[ref35] Horton D. Wohl R. R. (1956). Mass communication and Para-social interaction: observations on intimacy at a distance. Psychiatry 19, 215–229. doi: 10.1080/00332747.1956.11023049, 13359569

[ref36] Ivanov S. Soliman M. Tuomi A. Alkathiri N. A. Al-Alawi A. N. (2024). Drivers of generative AI adoption in higher education through the lens of the theory of planned behaviour. Technol. Soc. 77:102521. doi: 10.1016/j.techsoc.2024.102521

[ref37] Kock N. (2015). Common method bias in PLS-SEM: a full collinearity assessment approach. Int. J. e-Collab. 11, 1–10. doi: 10.4018/ijec.2015100101

[ref38] Ladwig G. Tanck J. A. Quittkat H. L. Vocks S. (2024). Risks and benefits of social media trends: the influence of “fitspiration,” “body positivity,” and text-based “body neutrality” on body dissatisfaction and affect in women with and without eating disorders. Body Image 50:101749. doi: 10.1016/j.bodyim.2024.101749 38850713

[ref39] Li H. Xu J. Luo Y. Wang C. L. (2025). The role of teachers’ direct and emotional mentoring in shaping undergraduates’ research aspirations: a social cognitive career theory perspective. Int. J. Mentor. Coach. Educ. 14, 123–142. doi: 10.1108/IJMCE-07-2023-0064

[ref40] Li Y. Xu J. Zhang X. Chen G. (2024). The relationship between exercise commitment and college students’ exercise adherence: the chained mediating role of exercise atmosphere and exercise self-efficacy. Acta Psychol. 246:104253. doi: 10.1016/j.actpsy.2024.104253, 38653082

[ref41] Lin Y. Niu R. Yu Z. (2023). Roles of ambiguity tolerance and learning effectiveness: structural equation modeling evidence from EFL students’ perceptions of factors influencing peer collaboration. Lang. Teach. Res. doi: 10.1177/13621688231216201

[ref42] Lin Y. Yu Z. (2025). Elucidating university students’ intentions to seek automated writing feedback from Grammarly: toward perceptual and systemic predictors. Humanit. Soc. Sci. Commun. 12:7. doi: 10.1057/s41599-024-03861-1

[ref43] Liu X. (2025). Fitness influencers in Chinese social media: role models for youth or unattainable ideals? Acta Psychol. 260:105743. doi: 10.1016/j.actpsy.2025.105743 41086746

[ref44] McAuley E. (1993). Self-efficacy and the maintenance of exercise participation in older adults. J. Behav. Med. 16, 103–113. doi: 10.1007/BF00844757, 8433355

[ref45] McKinley N. M. Hyde J. S. (1996). The objectified body consciousness scale: development and validation. Psychol. Women Q. 20, 181–215. doi: 10.1111/j.1471-6402.1996.tb00467.x

[ref46] Moffitt R. L. (2024). A psychosocial investigation of exercise preferences in real and virtual environments. Psychol. Sport Exerc. 70:102530. doi: 10.1016/j.psychsport.2023.102530, 37678040

[ref47] Newman-Beinart N. A. Norton S. Dowling D. Gavriloff D. Vari C. Weinman J. A. . (2017). The development and initial psychometric evaluation of a measure assessing adherence to prescribed exercise: the exercise adherence rating scale (EARS). Physiotherapy 103, 180–185. doi: 10.1016/j.physio.2016.11.001, 27913064

[ref48] Pappas I. O. Woodside A. G. (2021). Fuzzy-set qualitative comparative analysis (fsQCA): guidelines for research practice in information systems and marketing. Int. J. Inf. Manag. 58:102310. doi: 10.1016/j.ijinfomgt.2021.102310, 38826717

[ref49] Podsakoff P. M. MacKenzie S. B. Lee J.-Y. Podsakoff N. P. (2003). Common method biases in behavioral research: a critical review of the literature and recommended remedies. J. Appl. Psychol. 88, 879–903. doi: 10.1037/0021-9010.88.5.879, 14516251

[ref50] Porter C. D. Kwan M. Y. W. Meca A. Brown D. M. Y. (2024). Exercise identity and physical activity behavior during late adolescence: a four wave cross-lagged panel model. Psychol. Sport Exerc. 73:102641. doi: 10.1016/j.psychsport.2024.102641, 38593967

[ref51] Powell J. Pring T. (2024). The impact of social media influencers on health outcomes: systematic review. Soc. Sci. Med. 340:116472. doi: 10.1016/j.socscimed.2023.116472, 38070305

[ref52] Ragin C. C. (2008). Redesigning social Inquiry: Fuzzy sets and beyond. Chicago, IL: University of Chicago Press.

[ref53] Ragin C. C. (2009). “Qualitative comparative analysis using fuzzy sets (fsQCA),” in Configurational Comparative Methods: Qualitative Comparative Analysis (QCA) and Related Techniques, eds. Rihoux B. Ragin C. C. (Thousand Oaks, CA: SAGE Publications, Inc), 87–122.

[ref54] Ragin C. C. (2014). The Comparative Method: Moving beyond Qualitative and Quantitative Strategies. Berkeley, CA: University of California Press. 266.

[ref55] Rhodes R. E. Kaushal N. Quinlan A. (2016). Is physical activity a part of who I am? A review and meta-analysis of identity, schema and physical activity. Health Psychol. Rev. 10, 204–225. doi: 10.1080/17437199.2016.1143334, 26805431

[ref56] Rhodes R. E. Rebar A. L. Strachan S. (2025). Physical activity identity as an axis of dual process motivation and self-regulation processes: current evidence and future research directions. Psychol. Sport Exerc. 80:102923. doi: 10.1016/j.psychsport.2025.102923, 40541819

[ref57] Robinson L. Prichard I. Nikolaidis A. Drummond C. Drummond M. Tiggemann M. (2017). Idealised media images: the effect of fitspiration imagery on body satisfaction and exercise behaviour. Body Image 22, 65–71. doi: 10.1016/j.bodyim.2017.06.001, 28654826

[ref58] Rubin R. B. McHugh M. P. (1987). Development of parasocial interaction relationships. J. Broadcast. Electron. Media 31, 279–292. doi: 10.1080/08838158709386664

[ref59] Schaefer L. M. Thompson J. K. (2014). The development and validation of the physical appearance comparison scale-revised (PACS-R). Eat. Behav. 15, 209–217. doi: 10.1016/j.eatbeh.2014.01.001, 24854806

[ref60] Seekis V. Bradley G. L. Duffy A. L. (2020). Appearance-related social networking sites and body image in young women: testing an objectification-social comparison model. Psychol. Women Q. 44, 377–392. doi: 10.1177/0361684320920826

[ref61] Siegenthaler P. Aegerter T. Fahr A. (2023). A longitudinal study on the effects of parasocial relationships and breakups with characters of a health-related TV show on self-efficacy and exercise behavior: the case of the biggest loser. Commun. Sport 11, 744–769. doi: 10.1177/21674795211045039, 37426744 PMC10326891

[ref62] Strachan S. M. Brawley L. R. Spink K. S. Jung M. E. (2009). Strength of exercise identity and identity-exercise consistency: affective and social cognitive relationships. J. Health Psychol. 14, 1196–1206. doi: 10.1177/1359105309346340, 19858339

[ref63] Strachan S. M. Kullman S. M. Rhodes R. E. (2026). Building and strengthening physical activity identity: a theory-informed user-guide. Health Psychol. Rev. 20, 172–196. doi: 10.1080/17437199.2025.2550359, 40862679

[ref64] Teixeira P. J. Carraça E. V. Markland D. Silva M. N. Ryan R. M. (2012). Exercise, physical activity, and self-determination theory: a systematic review. Int. J. Behav. Nutr. Phys. Act. 9:78. doi: 10.1186/1479-5868-9-78, 22726453 PMC3441783

[ref65] Tiggemann M. Zaccardo M. (2015). “Exercise to be fit, not skinny”: the effect of fitspiration imagery on women's body image. Body Image 15, 61–67. doi: 10.1016/j.bodyim.2015.06.003, 26176993

[ref66] Wang C. Chen X. Hu Z. Jin S. Gu X. (2025a). Deconstructing university learners' adoption intention towards AIGC technology: a mixed-methods study using ChatGPT as an example. J. Comput. Assist. Learn. 41:e13117. doi: 10.1111/jcal.13117

[ref67] Wang C. Chen X. Yu T. (2025b). What drives learners’ behavioral intention to share knowledge on social media: evidence from a fuzzy-set qualitative comparative analysis (FsQCA). Curr. Psychol. 44, 8766–8780. doi: 10.1007/s12144-025-07813-z

[ref68] Wang C. Dai J. Zhu K. Yu T. Gu X. (2024). Understanding the continuance intention of college students toward new E-learning spaces based on an integrated model of the TAM and TTF. Int. J. Hum. Comput. Interact. 40, 8419–8432. doi: 10.1080/10447318.2023.2291609

[ref69] Wang H. Guo G. Yang M. Jiang S. Yang W. Zhao Y. (2026). Social media as a double-edged sword in sports nutrition dissemination: opportunities, challenges, and pathways forward. Current Nut. Rep. 15:14. doi: 10.1007/s13668-026-00735-7, 41733750

[ref70] Xia Y. Deng Y. Tao X. Zhang S. Wang C. (2024). Digital art exhibitions and psychological well-being in Chinese generation Z: an analysis based on the SOR framework. Hum. Soc. Sci. Commun. 11. doi: 10.1057/s41599-024-02718-x

[ref71] Yang J. W. (2025). How does fitness-related social media use influences exercise intention? A moderated mediation model within the theory of planned behavior framework. Acta Psychol. 261:105958. doi: 10.1016/j.actpsy.2025.105958, 41274016

[ref72] Yu T. Dai J. Chen X. Wang C. (2025). Factors influencing continuance intention in blended learning among business school students in China: based on grounded theory and FsQCA. Interact. Learn. Environ. 33, 1–28. doi: 10.1080/10494820.2024.2370477

[ref73] Yu T. Dai J. Wang C. (2023). Adoption of blended learning: Chinese university students’ perspectives. Hum. Soc. Sci. Commun. 10:390. doi: 10.1057/s41599-023-01904-7

