# Supplementary material for: Associations between fitness social media exposure and exercise adherence: dual pathways via parasocial relationships and body image pressure
Source: Front Psychol. 2026 Jul 2;17:1860956. doi: 10.3389/fpsyg.2026.1860956 (PMC13390499; doi:10.3389/fpsyg.2026.1860956)
Supplement: Supplementary file 1 [file Supplementary_file_1.docx]

Appendix 1 Questionnaire items.

| Variable | Item | Source |
| --- | --- | --- |
| Fitness Social Media Exposure (FSME) | FSME1: Over the past month, I have frequently browsed content related to fitness, training, or body shaping on social media. | Yang, 2025 |
|  | FSME2: Over the past month, fitness-related content has frequently appeared in my social media feed. |  |
|  | FSME3: Over the past month, I have actively searched for content related to fitness training, body shaping, or shared exercise experiences. |  |
|  | FSME4: Over the past month, I have often spent time watching fitness-related short videos, posts, or experience-sharing content. |  |
| Parasocial Relationship (PSR) | Instruction: Please first recall the fitness influencer or fitness account that you watched most frequently on social media over the past month. Please answer the following items based on that person/account. | Rubin & McHugh, 1987; Breves et al., 2025b |
|  | PSR1: I feel that I have come to know this fitness influencer’s style and characteristics quite well. |  |
|  | PSR2: When viewing their content, I feel as if I am interacting with them. |  |
|  | PSR3: I feel a sense of closeness to this fitness influencer. |  |
|  | PSR4: I feel that there is some kind of psychological connection between me and this fitness influencer. |  |
|  | PSR5: I look forward to this fitness influencer posting new content. |  |
| Exercise Self-Efficacy (ESE) | ESE1: Even when I am busy with study or work, I am confident that I can exercise as planned. | McAuley, 1993; Dwyer et al., 1998 |
|  | ESE2: Even when I feel tired or lack energy, I am confident that I can complete my planned exercise. |  |
|  | ESE3: Even when I am in a bad mood or under a lot of stress, I am confident that I can persist in completing my exercise. |  |
|  | ESE4: Even when no one is around to accompany or support me, I am confident that I can exercise on my own. |  |
|  | ESE5: Even when time, space, or other conditions are inconvenient, I am confident that I can find a way to keep exercising. |  |
| Exercise Identity (EI) | EI1: I see myself as a person who exercises regularly. | Anderson & Cychosz, 1994; Rhodes et al., 2025 |
|  | EI2: Exercise is part of how I define myself. |  |
|  | EI3: The identity of being “a person who exercises regularly” is consistent with how I see myself. |  |
|  | EI4: If I do not exercise for a long time, I would feel that this is not the real me. |  |
| Physical Appearance Comparison (PAC) | PAC1: When browsing fitness-related social media content, I compare my appearance or body shape with that of others. | Schaefer & Thompson, 2014 |
|  | PAC2: When I see others’ results in fat loss, muscle gain, or body shaping, I evaluate whether I have reached a similar level. |  |
|  | PAC3: When browsing fitness content, I pay particular attention to the differences between my body and others’ in certain body parts. |  |
| Body Surveillance (BS) | BS1: In daily life, I often think about how my body looks. | McKinley & Hyde, 1996 |
|  | BS2: Compared with how my body feels, I pay more attention to how my body looks. |  |
|  | BS3: I view my body from an appearance-based perspective. |  |
|  | BS4: I often pay attention to whether my body shape or appearance matches my ideal state. |  |
| Social Physique Anxiety (SPA) | SPA1: I feel uncomfortable when others may notice my body shape. | Hart et al., 1989 |
|  | SPA2: I worry that others may think that my body shape, weight, or muscle definition is not ideal enough. |  |
|  | SPA3: When wearing sportswear, tight-fitting workout clothes, or other close-fitting clothing, I feel nervous about my physical appearance. |  |
|  | SPA4: In gyms, sports fields, or other public exercise settings, I worry that others may make negative judgments about my body shape. |  |
| Exercise Adherence (EA) | EA1: Over the past month, I have basically been able to exercise according to my originally planned frequency. | Newman-Beinart et al., 2017 |
|  | EA2: Over the past month, the amount of exercise I actually completed was generally consistent with my original plan. |  |
|  | EA3: Over the past month, I was able to incorporate exercise into my daily life relatively consistently. |  |
|  | EA4: Over the past month, I rarely postponed or canceled exercise that I had originally planned. |  |
